# Modified less invasive anterior subcutaneous fixator for unstable Tile-C-pelvic ring fractures: a biomechanical study

**DOI:** 10.1186/s12938-019-0648-z

**Published:** 2019-03-29

**Authors:** Christopher A. Becker, Christian Kammerlander, Adrian Cavalcanti Kußmaul, Matthias Woiczinski, Christoph Thorwächter, Christian Zeckey, Fabian Sommer, Christoph Linhart, Simon Weidert, Eduardo M. Suero, Wolfgang Böcker, Axel Greiner

**Affiliations:** 1 Department of General Trauma & Reconstructive Surgery, University Hospital, LMU Munich, Munich, Germany; 2 Department of Orthopedics, Physical Medicine and Rehabilitation, University Hospital, LMU Munich, Munich, Germany

**Keywords:** Pelvic ring fracture, Biomechanical study, Tile-C-fracture, Minimally invasive fixation

## Abstract

**Background:**

Operative procedures for unstable pelvic ring fractures remain controversially discussed. Minimally invasive treatment options for pelvic ring fractures have several benefits for the patient. But they can also provide disadvantages. Anterior subcutaneous pelvic fixation (INFIX) has shown promising biomechanical results in pelvic ring fractures, but there is a high complication rate of nerve injuries. An additional screw to the INFIX seems to be more stable. The aim of this study is to compare biomechanical stability of a new modified unilateral INFIX fixing the unilateral injured pelvic ring with the standard INFIX.

**Methods:**

24 composite synthetic full pelvises were used in this study. 4 groups each with a number of six pelvic specimens were randomly assigned. A C1.3-type pelvic fracture was made with an osteotomy of the sacrum and an osteotomy of the anterior pelvic ring. Fracture fixation was performed within the four groups: (1) unilateral INFIX, (2) “extended” unilateral INFIX + additional pubic ramus pedicle screw, (3) bilateral INFIX, (4) “extended” bilateral INFIX + additional pubic ramus pedicle screw. All specimens were cyclic loaded with 200 N until maximum of 300 N. Distance/dislocation of the fracture fragments were detected with 3D-ultrasound measuring system. Stiffness was calculated.

**Results:**

Extended unilateral INFIX showed the lowest mean dislocation. Lowest rotational stability was displayed by the standard bilateral INFIX. A significant difference (P = 0.04) was shown between the extended unilateral INFIX and the “standard” bilateral INFIX in terms of rotational stability. Extended unilateral INFIX showed significantly improved stability of anterior fracture dislocation (P = 0.01) and unilateral INFIX showed the highest rotational stiffness. Anterior fixation stiffness of the unilateral INFIX was significantly improved using an additional symphysis/pubic ramus screw (P = 0.002).

**Conclusion:**

Extended unilateral INFIX (+ additional pubic ramus pedicle screw) is a feasible minimally invasive treatment for anterior pelvic ring fractures. Higher stability and lower probability of bilateral nerve damage is provided by the extended unilateral INFIX compared to the standard bilateral INFIX.

## Introduction

While consensus exists on the need of surgical treatment of unstable pelvic ring fractures, the choice of the ideal strategy for osteosynthesis remains controversial [1–3]. Current clinical research shows that minimally invasive strategies may have several benefits for patients compared to the more invasive plate osteosynthesis [4, 5]. The anterior subcutaneous pelvic fixator (INFIX) has formerly been described as a treatment option for unstable pelvic ring fractures and biomechanical studies reveal a superior stability of the INFIX compared to a supraacetabular external fixator [6]. Clinical data show that the stability and the clinical outcome of patients with pelvic ring fracture treated with an INFIX (or INFIX with three screws) is sufficient compared to standard open plate osteosynthesis [7].

Nonetheless, some clinical data also hints towards increased complication rates for the INFIX as well as supraacetabular external fixation, the main complication being damage to the superficial femoral cutaneous nerve [8, 9]. This nerve is at risk on both sides of the pelvic ring as it is situated closely to the lateral screw and it can easily be crushed between the bone cortex and the rod.

The configuration providing optimal stability when using the INFIX is debatable. A very limited number of papers demonstrated that it might be useful to add a third screw next to the pubic symphysis [7]. Another option to improve stability while reducing the risk of nerve damage on the contralateral side could be the connection between the unilateral supraacetabular screw with two screws on both side of the pubic symphysis.

The purpose of this study was to evaluate the stability of the unilateral INFIX compared to a bilateral INFIX with two variations of each. We hypothesize that both configurations offer a comparable stability and stiffness of a unilateral and a bilateral INFIX, rendering the unilateral INFIX a viable option for unstable pelvic ring fractures.

## Methods

**Fig. 1:**
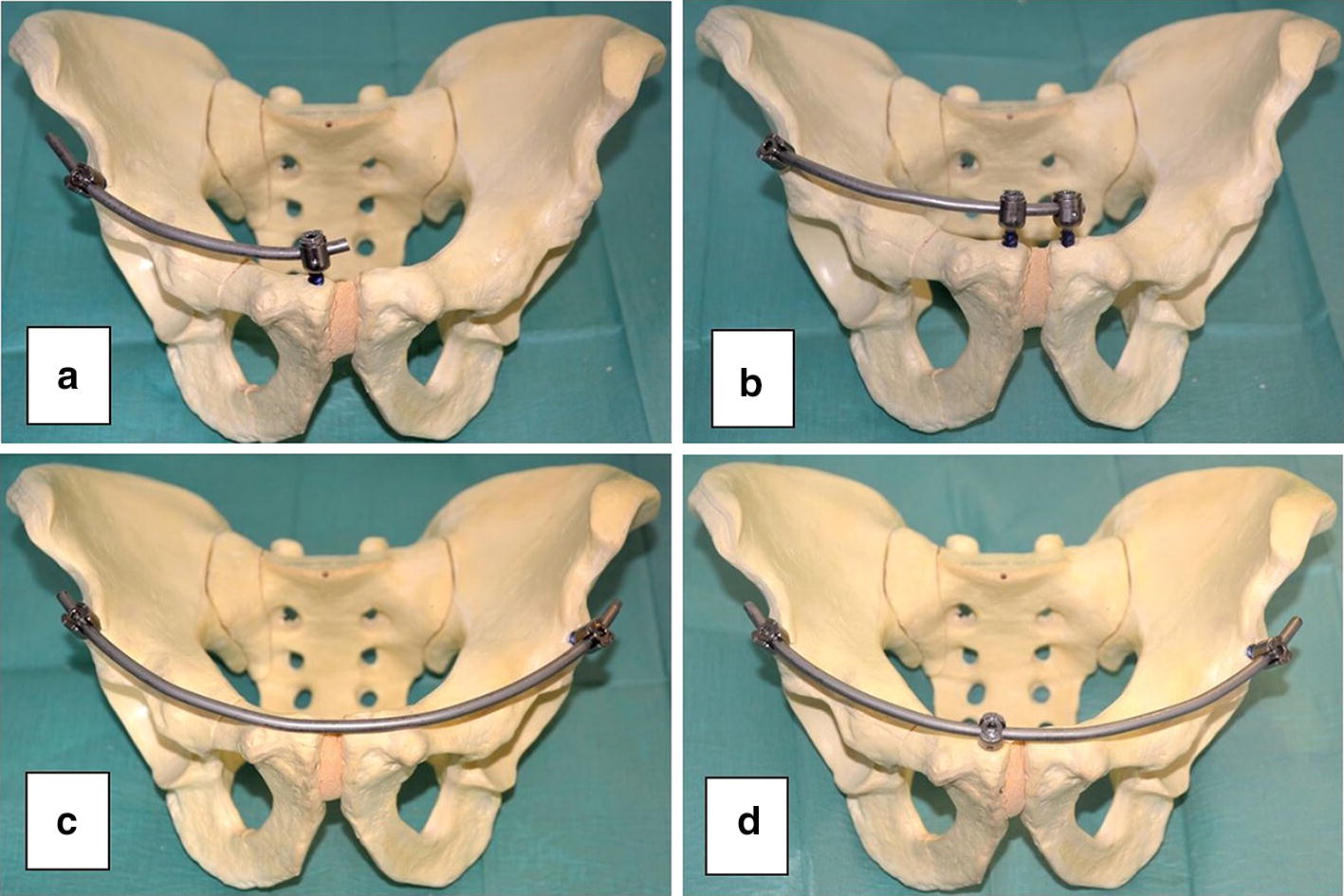
**a** Unilateral INFIX + 2 iliosacral screws, **b** extended unilateral INFIX + 2 iliosacral screws, **c** bilateral INFIX + 2 iliosacral screws, **d** extended bilateral INFIX + 2 iliosacral screws

24 composite synthetic full pelvises (Model: Full Pelvis 1301, Sawbones^®^; Pacific Research Laboratories, Vashon, WA, USA) were used in this study. Each specimen was randomly assigned to one of four fixation groups each, yielding six pelvic specimens per each of the following groups: unilateral INFIX (Group 1); “extended” unilateral INFIX + additional pubic ramus pedicle screw (Group 2); bilateral INFIX (Group 3); “extended” bilateral INFIX + additional pubic ramus pedicle screw (Group 4) (Fig. 1).

After randomization, an AO Classification type C1.3 pelvic ring fracture was simulated in each specimen by performing an osteotomy of the sacrum (lateral of the foramen, Denis I-type) and an osteotomy of the anterior pelvic ring (complete fracture of pubic ramus and ischium) using a hacksaw.

Once the fracture models were created, INFIX fixation was applied using two or three pedicle screws (6.5 × 50 mm, Viper, Synthes, USA) and a connection rod (cobalt chrome 5.5 mm, Synthes, USA) depending on the osteosynthesis in the anterior inferior iliac spine and symphyseal part of the ramus ossis pubis uni- or bilateral. In every group the SI-joint was unilaterally fixed with two cannulated iliosacral screws (7.5 × 70 mm, Königsee Implantate GmbH, Allendorf, Germany) placed into S1.

Tension banding of the iliotibial tract is simulated with the use of cable pulls fixed ipsilateral to the iliac crest.

Our biomechanical test protocol was conducted according to McDonald et al. [10]. A bipolar hemi-prosthesis was inserted into the acetabulum in order to simulate axial skeletal loading.

Fracture fragment displacement were measured using a 3D ultrasound tracking system with an error margin of 0.1 mm (Zebris CMS20; Zebris Medical GmbH, Isny, Germany). A total of three sensors were fixed onto the acetabulum, the symphysis and the sacrum.

**Fig. 2:**
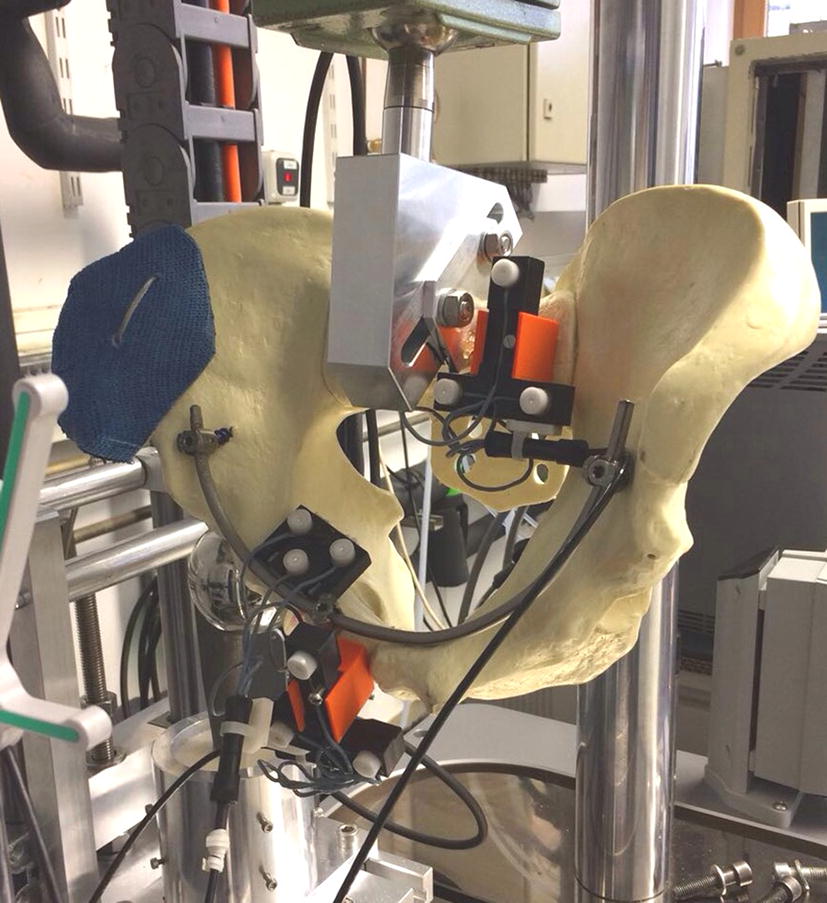
Biomechanical setup with the pelvis fixed onto the testing machine. Sensors were fixed at the sacrum, the pubic symphysis and the acetabulum

An all-electric industrial loading machine (ElectroPulsTM E10000 Linear-Torsion) was used in this study with the following test protocol: (1) axial loading up to 200 N; (2) loading to 150 N for 30 s; (3) cyclic loading of 25 cycles with a frequency of 0.25 Hz between 100 and 200 N; (4) loading to 150 N for 30 s; (5) maximal loading up to a force of 300 N or until reaching a displacement limit of − 28 mm; (6) system back to its original position of + 28 mm. Due to the lower load capacity of the synthetic pelvic models, a force of a maximum of 300 N (approximately 1/2 body weight) was used for the cyclical tests in order not to jeopardize the integrity of the osteosynthesis. This should be taken into account when interpreting the results of the current study and for any in vivo considerations. The relative distance of each the three sensors to each other was recorded. A maximum distance of the sensors was defined as the maximum displacement of the fragments in a certain direction (mm). Stiffness was defined as the loading force divided by the maximum displacement distance (N/mm) (Fig. 2).

### Statistical analysis

We used linear regression to test for an effect of fixation techniques on fracture fragment displacement and construct stiffness. Robust standard errors were calculated to decrease to chance of a type I error. Pairwise comparisons were performed between techniques and a Holm correction was applied to all P-values. Alpha was set to 0.05 for all tests. Descriptive statistics are presented as mean and standard deviation or 95% confidence interval (95% CI) wherever appropriate. All testes were conducted using R version 3.5.1 (R Foundation for Statistical Computing, Vienna, Austria).

## Results

### Displacement

Mean displacement (distance Symphysis —Sacrum) was 7.4 ± 2.9 mm and mean anterior displacement was 3.6 ± 1.1 mm.

**Fig. 3:**
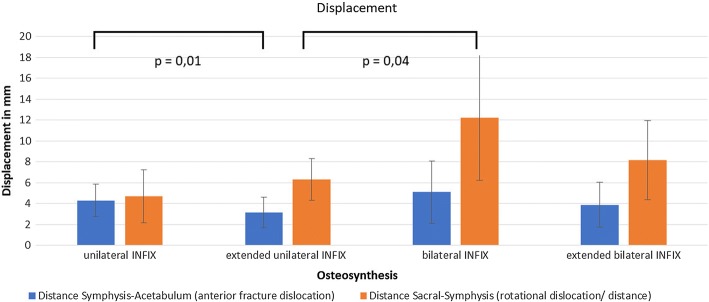
Average displacement (mm) of the four different groups: (1) unilateral INFIX, (2) extended unilateral INFIX, (3) bilateral INFIX, (4) extended bilateral INFIX

Extended unilateral INFIX showed the lowest mean displacement. The highest rotational stability was displayed by both the unilateral INFIX as well as the extended unilateral INFIX. The lowest rotational stability was displayed by the classic bilateral INFIX (Fig. 3).

A significant difference (P = 0.004) was shown between the extended unilateral INFIX and the “standard” bilateral INFIX in terms of rotational stability.

Additional symphysis/pubic ramus pedicle screws installed to the unilateral INFIX (= extended unilateral INFIX) showed significantly improved stability of anterior fracture dislocation (P = 0.01).

Anterior stability of the fracture of the bilateral INFIX is slightly, not significantly improved by an additional symphysis/pubic ramus pedicle screw (P = 0.7).

### Stiffness

**Fig. 4:**
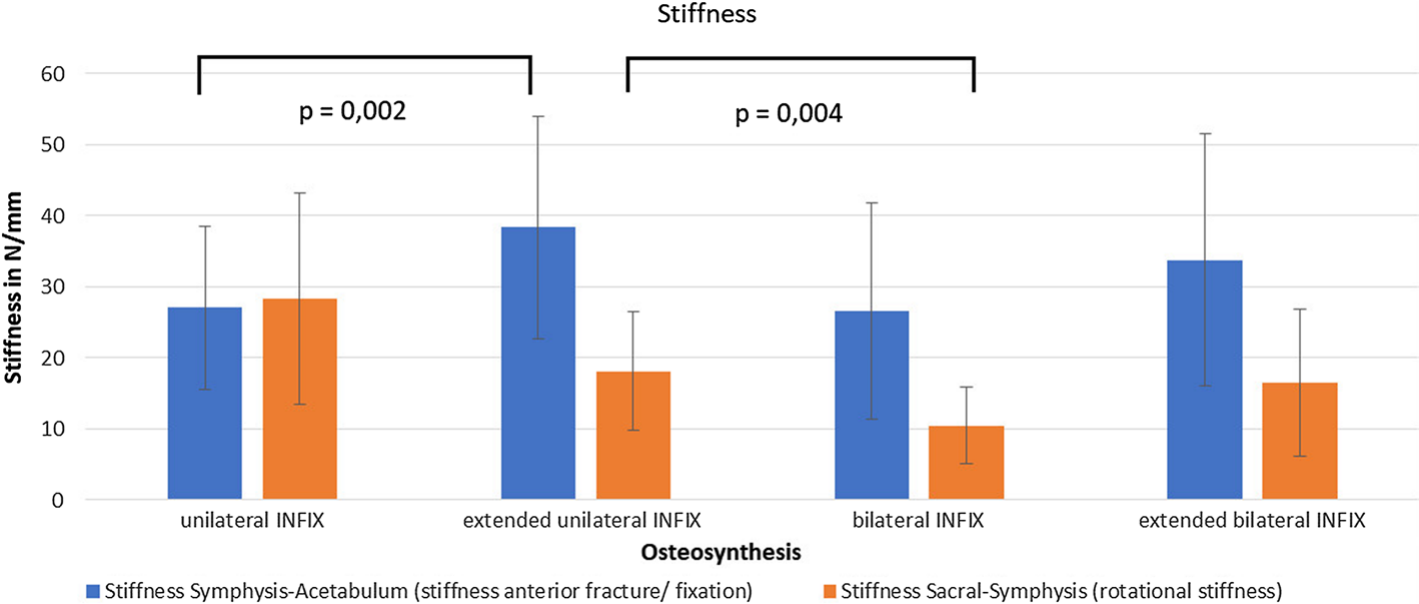
Mean stiffness (N/mm) of the four different groups: (1) unilateral INFIX, (2) extended unilateral INFIX, (3) bilateral INFIX, (4) extended bilateral INFIX

Mean rotational stiffness (Stiffness Symphysis —Sacrum) was 48.7 ± 17.5 N/mm and the mean anterior fracture/fixation stiffness was 84.6 ± 21.5 N/mm (Fig. 4).

The highest rotational stiffness was seen with the unilateral INFIX. There was also a significant difference (P = 0.004) of the unilateral INFIX with an additional symphysis/pubic ramus pedicle screw compared with the bilateral INFIX.

Anterior fixation stiffness of the unilateral INFIX was significantly improved using an additional symphysis/pubic ramus screw (P = 0.002).

## Discussion

Optimal fixation strategy in unstable pelvic fractures remains controversial topic in orthopaedic trauma care. In the last decade, minimally invasive approaches have shown promising results. The aim of the current study was to evaluate the biomechanical stability of a unilateral and extended unilateral INFIX compared to the a bilateral INFIX for fixation and stabilization of unstable pelvic fractures. The main findings of the current study were that. The biomechanical stability of the extended unilateral INFIX was significantly superior to that of the bilateral INFIX. He addition of a pubic ramus or symphysis pedicle screw to the unilateral INFIX provided significantly better anterior stiffness and decreased anterior fracture fragment displacement.

These results show that, in cases of unilateral pelvic ring instability, the classic bilateral INFIX could be replaced by the extended unilateral INFIX, reducing the risk of nerve damage on the contralateral, uninjured site. Furthermore, we see the potential for using the additional pubic ramus pedicle screw for reduction of the fracture, as it offers good control of the medial fragment. Due to the three-point-attachment, the rod could play a role in reducing the intermediate fragment attached to the screw in the middle, similar to spine surgeries.

Preliminary results of ongoing biomechanical have demonstrated good biomechanical stability of a modified INFIX for acetabular T-type-fractures, simultaneously using this device for the reduction of the anterior acetabular column.

Displaced fractures of the anterior pelvic ring often require open reduction and internal fixation. For this procedure, anterior approaches such as the modified Stoppa approach can be used [11]. These approaches have many disadvantages, such as high blood loss and the risk of nerve damage due to traction [11–13].

Minimally invasive treatment options of the anterior pelvic ring are normally performed with a retrograde transpubic screw, a supraacetabular external fixator or, as a new method, with the INFIX. These treatments also have some disadvantages, mainly regarding difficulties to achieve a proper reduction in displaced fractures. An additional supraacetabular external fixator may help to reduce the fracture but may introduce a higher risk of infection of the pin screws, loosening of the screws, loss of reduction, as well as a massive discomfort of the patient who has to cope with this construction in his or her daily routine [14, 15]. The INFIX also seems more effective than external fixation at reducing postoperative surgical site pain [16]. Other disadvantages of the external pelvic fixator include worse outcomes in diabetic and obese patients [17].

Biomechanically, the INFIX is more stable compared to the external fixator and provides better stability [6]. The symphysis-fixed INFIX (extended unilateral INFIX) could provide improved rotational stability compared to the bilateral INFIX with fixation of the symphysis. A finite element study by Song et al. [17] showed better rotational stability of the plate fixation fixed to the symphysis compared to the bilateral INFIX. It was concluded that this could be due to the fixation to the symphysis [17].

Our results also underline that the extended unilateral INFIX could provide better rotational stability than the standard bilateral INFIX, possibly because fixation next to the symphysis provides more anterior stability when using the INFIX for anterior pelvic ring fractures. Further biomechanical studies should follow especially in human pelves to confirm these findings.

We assume that the extended unilateral INFIX has better stability than the bilateral INFIX with the possible advantage of performing a reduction of the fracture with the pre-bend rod.

A problem of the extended INFIX, and also the classic INFIX, is the anterior fixation near the pubic symphysis. Anatomically, the anterior fixation site is located near the urinary bladder, therefore having the potential for irritating it, especially when filled. The potential bladder damage is also a problem when performing INFIX or extended INFIX [2]. In clinical situations, another problem of the INFIX is the potential damage to nerve and vessel structures like the lateral femoral cutaneous nerve, the femoral artery or the femoral vein during the operation. On the other hand, the more invasive Stoppa approach for ORIF is more challenging for the surgeon and has high potential for damaging blood vessel structures or the peritoneum [12].

Our study has a number of potential limitations. The composite bones do not fully resemble the biomechanical conditions in a human body, but they do have the advantage of providing reproducible and comparable biomechanical testing, thus avoiding the variability inherent to cadaveric specimens. In terms of fracture reduction, the synthetic bones are by far easier to reduce than human in vivo pelvic bones. However, previous studies have already demonstrated that the INFIX is a feasible technique of reduction and fixation of fractures in patients with pelvic trauma.

## Conclusion

The extended unilateral INFIX with an additional pubic ramus pedicle screw is a feasible minimally invasive alternative treatment for anterior pelvic ring fractures, especially in Tile-C unstable pelvic fractures. Further in vivo studies are warranted to evaluate the use of the extended unilateral INFIX in anterior pelvic ring fractures in a clinical setting.

## Abbreviation

INFIX: internal fixator
